# Decreased phrenic nerve compound muscle action potential, inspiratory muscle strength, and exercise capacity after COVID-19

**DOI:** 10.3389/fneur.2023.1308443

**Published:** 2024-01-16

**Authors:** Karin Vonbank, Helena Nics, Ralf Harun Zwick, Julia Maasz, Benjamin Sabic, Marijan Potzmann, Georg Brandhofer, Julia Fuchs, Lusine Yeghiazaryan, Martin Burtscher, Tatjana Paternostro-Sluga

**Affiliations:** ^1^Klinik Pirawarth in Wien, Vienna, Austria; ^2^Medical University of Vienna, Vienna, Austria; ^3^Department of Physical Medicine and Rehabilitation, Klinik Floridsdorf, Vienna, Austria; ^4^Ludwig Boltzmann Institute for Rehabilitation Research, Therme Wien Med, Vienna, Austria; ^5^Medical University of Vienna, Center for Medical Data Science, Institute of Medical Statistics, Vienna, Austria; ^6^Department of Sport Science, University of Innsbruck, Innsbruck, Austria

**Keywords:** COVID-19 infection, exercise capacity, phrenic nerve, inspiratory muscle weakness, maximum inspiratory pressure

## Abstract

**Objectives:**

Respiratory muscle weakness with higher ventilatory demands were reported even in patients recovering from only mild COVID-19 symptoms. Aim of this study was to assess the function of phrenic nerve and inspiratory respiratory muscle as well as cardiopulmonary exercise capacity in patients with prolonged exertional dyspnea after COVID-19 infection.

**Methods:**

In this observational exploratory study, electrophysiological examination of the phrenic nerve, inspiratory muscle capacity as well as lung function test, 6-min walk distance (6MWD) and cardiopulmonary exercise test, have been performed in 22 patients post COVID-19 diagnosis (post-CoV).

**Results:**

Exercise capacity (peak workload, Wpeak % predicted and peak oxygen uptake, VO_2_peak % predicted) were significantly affected in the post-CoV patients (61.8 ± 23.3 Wpeak % and 70.9 ± 22.3 VO_2_peak %). Maximum inspiratory pressure (MIP) was reduced (60.1 ± 25.5 mbar). In 6 of the 22 patients the electrophysiological response of the phrenic nerve was pathologically decreased (reduced compound muscle action potential, CMAP), while nerve conduction velocity (NCV) was normal, which corresponds to reduced muscle fiber contraction capacity. Positive relationships were demonstrated between 6MWD and MIP (*r*_*s*_ = 0.88) as well as quality of life questionnaire (CRQ) and MIP (*r*_*s*_ = 0.71) only in patients with reduced CMAP.

**Discussion:**

Respiratory muscle weakness and exercise capacity is associated with reduced phrenic nerve CMAP without signs of neuropathy. This indicates that muscle fiber pathology of the diaphragm may be one pathophysiological factor for the prolonged respiratory symptoms after COVID-19 infections.

## Introduction

Coronavirus disease 2019 (COVID-19) is a global pandemic affecting individuals to varying degrees, ranging from a few days of mild symptoms to acute respiratory distress syndrome (ARDS) requiring ICU treatment, including ventilation support, and death (1, 2). Most severe acute respiratory syndrome coronavirus type 2 (SARS-CoV-2) infections were mild to moderate (3–5). However, it has been suggested that even patients with mild symptoms can suffer from prolonged physical impairment and skeletal limitation with impaired VO_2_peak (6). Neurological and musculoskeletal abnormalities with pain and weakness in lower limbs (7–9) as well as respiratory muscle weakness with higher ventilatory demands were reported even in patients recovering from only mild COVID-19 symptoms (10). At 4–7 months after onset of COVID 19 infection in hospitalized patients, the most common symptoms reported were dyspnea, fatigue and muscle weakness, seen in approximately 53–64% of patients (11–13), as well as headache (18% of patients) (14, 15) whereas dyspnea was not explained by long-term pulmonary abnormalities (11). Due to the heterogeneity of the clinical presentation the muscle-related symptoms are likely to be multifactorial, such as systemic inflammation and immune cell infiltration, hypoxia, adverse effects of medication and muscle disuse (16). Possible risk factors are acute myopathy during the acute viral illness, or nerve function impairment. Acute inflammatory sensory and motor polyradiculoneuropathy or Guillain-Barre syndrome were described in patients post COVID infection (17). Decreased muscle membrane excitability, slowing of nerve conduction velocity and axonal degeneration with prolonged duration of the compound muscle action potential are characteristics of critical illness neuromyopathy (18, 19). The impact of these neuromyopathological changes of respiratory muscles in patients with COVID-19 infection is still unclear.

The aims of this observational study were (1) to assess electrophysiological examinations of the phrenic nerve, and (2) to evaluate inspiratory muscle capacity and cardiopulmonary exercise capacity in adult post COVID-19 patients with prolonged exertional dyspnea.

## Methods

### Subjects

A total of 22 adult patients after COVID-19 infection were enrolled in this prospective, exploratory trial (between April 2021 and August 2021) referred to the rehabilitation outpatient center Klinik Pirawarth in Wien and Therme Wien Med, who accepted electrophysiological examination of the phrenic nerve.

All patients underwent electrophysiological examinations of the phrenic nerve, measurement of the maximum inspiratory pressure (MIP), lung function testing, 6-min walking distance (6-MWD) and symptom-limited cardiopulmonary exercise test (CPET) prior to a multidisciplinary comprehensive rehabilitation program. The rehabilitation program consisted of 60 training sessions, each session 45 min, according to the guidelines of the Austrian insurance program PVA (3 times per week over a period of 6 weeks: endurance, strength, and inspiratory muscle training, education programs).

The study protocol was approved by the Ethics committee of the medical university of Vienna (1227/2022) and is in accordance with the declaration of Helsinki.

### Electrophysiological examination

**Figure 1:**
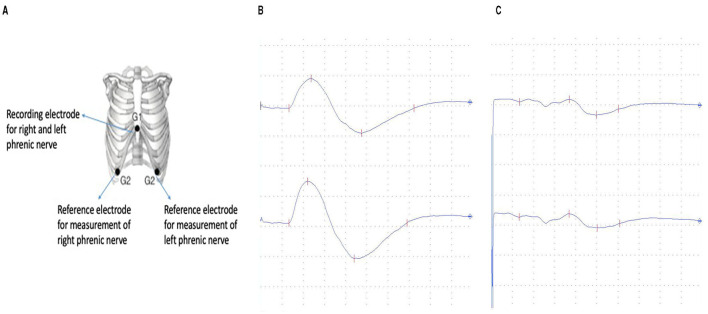
**(A)** Electrode position for the electophysiological examination of the phrenic nerve (20). **(B)** Normal phrenic nerve compound muscle action potential (1280 μV) with normal latency (6.6 ms). **(C)** Pathological decreased compound muscle action potential (260 μV) with normal latency (6.6 ms).

The electrophysiological examinations of the phrenic nerve were performed using a Keypoint EMG device (Medtronic, Dantec Medical A/S, Skovlunde, Denmark). The participants underwent the examination in supine position without cushioning underneath their heads providing a standardized position. The recording electrode was applied 50 mm cranial of the xyphoid process which suffices for both left- and right-sided phrenic nerve stimulation (Figure 1A). The reference electrode was applied on the right and the left side of the thorax within the 7th intercostal space at the height of the anterior axillary line. A ground electrode was placed on the sternum cranial to the recording electrode keeping a distance of at least 20 mm. Additionally, an earth strap was wrapped around the left upper arm of the probands. For the stimulation of the phrenic nerve a surface electrode was used.

Providing precise stimulation of the nerve the stimulator was positioned at the posterior border of the sternocleidomastoid muscle at the height of the cricoid cartilage. The participants were asked to turn their heads to the contralateral side of stimulation and perform a contraction of the sternocleidomastoid muscle. After the correct position was identified the probands turned their heads back to neutral position (20, 21).

The duration of the stimulation impulse was standardized at 0.2 ms. For supramaximal stimulation, the intensity of the impulse was increased in intervals of 10 mA until the recorded motor potential no longer increased. To ensure no superimposition occurred owing to a motor potential of the brachial plexus maximum stimulation was performed with 70 mA. After supramaximal stimulation was identified, the patient was asked to inhale and hold the breath until the next supramaximal stimulation. Since prior studies described the compound muscle action potential (CMAP) being of larger size during inspiration than during expiration.

The latency was defined as the time of stimulation to the negative deflection of the CMAP of the diaphragm. Computer-generated latency markers were corrected manually by the investigators if necessary. CMAP was measured peak to peak. Instrumental Parameters were set to an amplification of 500 μV/D and a sweep speed of 3 ms/D. Normative data was used as stated in Vincent et al. with a lower limit for the CMAP at 280 μV for men and 250 μV for women and an upper limit for the latency at 8.56 ms for men and 8.41 ms for women.

### Pulmonary lung function test

Lung function was assessed by the use of body plethysmography and spirometry.

Predicted normal values were derived from the reference values in accordance with current recommendations (22). The following resting pulmonary function parameters were determined: vital and forced vital capacity (VC and FVC), forced expiratory volume in one second (FEV1), FEV1/FVC, total lung capacity (TLC), residual volume (RV). Diffusing capacity for carbon monoxide (DLCO) and the CO transfer factor (KCO, DLCO/VA) were measured by single-breath technique. Results were expressed as absolute values and percent of predicted values. Each value represents the best of at least three measurements. Spirometry, whole- body plethysmography including DLCO were performed with the Master Screen Body (FA Reiner/Viasys, Carefusion, Australia).

### Quality of life questionnaire

The chronic respiratory disease questionnaire (CRQ) was evaluated before and at the end of the rehabilitation to assess the quality of life (in our patients) consisting of 20 items including 4 different sections (dyspnea, fatigue, emotional function and mastery). Items in each section are scored from 1 (most severe) to 7 (no impairment). Total scores are reported.

### Cardiopulmonary exercise test

All participants underwent a symptom-limited CPET on an Ergoline 800 bicycle (Sensormedics, United States) with respiratory gas-exchange analysis, using a step protocol with progressive increase in workload every minute according to a total exercise time between 8 and 12 min. The increment was adapted to the expected maximum working capacity. Patients were encouraged to exercise until exhaustion. A cycling frequency of 60–80 revolutions per minute (rpm) had to be maintained. Patients were encouraged to exercise until exhaustion. The test was stopped when the subject failed to maintain a pedal frequency of at least 60 rpm. Blood pressure was measured every 2 min and continuous 12-lead electrocardiogram and peripheral oxygen saturation (SpO_2_) were recorded. Breath-by-breath minute ventilation (VE), carbon dioxide output (VCO_2_) and oxygen uptake (VO_2_) were measured using the Sensormedics 2900 Metabolic Measurement Cart. Blood gas analysis was performed at rest and at peak exercise (ABL800 Flex, Drott Medizintechnik GmbH, Austria).

### 6-min walking distance

The 6MWD was completed as recommended by the American Thoracic Society (23) and Butland et al. (24). 6MWD was applied in a 30-m unobstructed corridor. Patients were instructed to walk their own pace but to cover as much meter as possible within 6 min. Each minute standardized encouragement was given to the patients. Patients were allowed to stop and rest during test but were instructed to go on walking as soon as they were able to do so. Heart rate and oxygen saturation was monitored during the test. Maximum heart rate values achieved during the tests were recorded. Modified Borg Dyspnea Scale was used before and after the 6MWD. Walking distance in meters has been evaluated (25).

### Respiratory muscle strength

Measurements of MIP were determined in a sitting position and the nostrils occluded by a clip (Respifit S device, Biegler, Mauerbach, Austria/AstraPEP). After exploration and demonstration of the maneuver, the patient was encouraged to exhale slowly to the level of functional residual capacity and then urged to inhale with as much force as possible and to sustain the pressure for at least 1 s. Each subject had to perform 10 attempts to determine MIP. The length of the break between each maneuver was 10 s. The peak values of MIP were measured in mbar.

### Statistical analysis

Statistical analyses were performed using IBM SPSS version 28.0 (IBM SPSS Statistics for Windows, Chicago, IL, USA) and R version 4.0.3. A descriptive data analysis was performed. Continuous variables were presented as mean ± SD (standard deviation), median (interquartile range, IQR) values and categorical variables were presented as numbers (percentages, %). To compare differences between patients with and without decreased CMAP (NCS positive vs. NCS negative) and increased latency and for comparison of severity differences for the continuous variables such as listed in **Table 2**, the Mann–Whitney *U*-test was calculated. For the categorical variables, such as gender Fisher‘s exact test was calculated. To evaluate associations between variables Spearman's rang correlaton (*r*_*s*_) test were used. The p-values are interpreted descriptively and no adjustment for multiple testing was performed.

All tests were considered statistically significant at *p* < 0.05.

## Results

### Baseline characteristics

All patients reported exertional dyspnea, 19 patients reported reduced exercise performance and four patients suffered neuropathic pain. Six out of 22 patients required hospitalization during the ongoing infection. The total study cohort consisted of 41 % female (nine patients) and 59 % male (13 patients) individuals. The mean age of the total cohort was 48 (12.4) years; mean height was 175.1 (9.5) cm and mean weight 83.9 (22.7) kg. Baseline characteristics and lung function parameters are shown in Table 1. TLC was 94.9 (16.4) % predicted, PEF 88.8 (28.4) % pred and FVC in the total study group was 85.3 (19.4) % pred.

**Table 1:** Baseline characteristics and lung function parameters.

**Subjects *n* (%)**	**22 (100)**
Female	9 (40.9)
Male	13 (59.1)
Age years	48.1 (12.4)
Weight kg	83.9 (22.7)
Height cm	175.1 (9.5)
BMI kg/m^2^	27.4 (7.1)
FEV1 liter	3.1 (1.0)
FEV1 %pred	85.3 (22.5)
FEV1/FVC %	79.4 (12.2)
TLC L	6.1 (1.4)
TLC %	94.9 (16.4)
PEF L	7.13 (2.5)
PEF %	88.8 (28.4)
FVC L	3.9 (1.1)
FVC %	85.3 (19.4)

BMI, body mass index; FEV 1, forced expired volume in 1 s; FVC, forced vital capacity; TLC, total lung capacity; PEF, peak expiratory flow volume.

### Electrophysiological examination of the phrenic nerve

Complete data have been collected for 22 patients. Two patients had to be excluded, as in one patient the phrenic nerve was not measurable due to technical difficulties, and one patient did not endure supramaximal stimulation which results in amplitudes below normal.

Within those 20 patients, 19 presented latencies within normative values (Figure 1B). One patient who reported to be long-term diabetic showed a prolonged latency of 12 ms for the right phrenic nerve and 8.6 ms for the left. Median values for right phrenic nerve in normal patients were 7.2 ms and 7.1 for the left side. The average side difference was 0.4 ms in the normative group.

Six patients did not reach normative values for the CMAP of the phrenic nerve (Figure 1C). Mean value in probands out of norm was 200 μV for the right phrenic nerve and 218 μV for the left side with a mean difference between left and right of 90 μV. Among the patients with CMAPs within normative values the mean was 821 μV for the right side and 836 μV for the left with an average side difference of 272 μV.

### Maximal exercise parameters and 6-min walking distance

Wpeak in the whole study group was 102.2 (44.3) Watt, 61.8 (23.3) % pred, the VO_2_peak was 19.0 (7.1) ml/kg/min; 70.9 (22.3) % pred. The 6MWD in the whole study group was 402.0 (130.9) meters.

### Lung function parameters, respiratory muscle force, and exercise capacity in patients without decreased phrenic nerve CMAP (NCS negative) and with decreased phrenic nerve CMAP (NCS positive)

There were no differences between men and women who were nerve conduction study (NCS) positive and negative. No significant differences could be found concerning the lung function parameters between both groups, but TLC, PEF and FEV1/FVC were slightly lower in the NCS positive group compared to the NCS negative group (Table 2). Also, MIP, the CRQ score, Wpeak and VO_2_peak were all slightly lower in the NCS positive group, but differences were not statistically significant.

**Table 2:** Differences in lung function and exercise capacity concerning phrenic nerve compound muscle action potential.

	**Mean (SD)**	**Mean (SD)**	
	**NCS negative**	**NCS positive**	***P*** **-value** ^*^
	***n*** = **16**	***n*** = **6**	
VO_2_peak (ml/min)	19.5 (6.7)	17.7 (9.0)	0.35
VO_2_peak (%pred)	74.5 (21.3)	61.8 (24.5)	0.37
Wpeak (Watt)	105.0 (39.8)	94.7 (58.2)	0.51
Wpeak (%pred)	63.7 (21.1)	56.8 (30.1)	0.61
6MWD (m)	403.8 (138.3)	397.0 (120.4)	0.86
MIP (mbar)	63.4 (25.1)	51.3 (26.7)	0.34
TLC (%pred)	98.1 (11.7)	86.4 (24.8)	0.22
FVC (%pred)	87.9 (15.0)	78.3 (28.8)	0.64
PEF (%pred)	91.5 (25.3)	81.7 (37.1)	0.54
PEF (L)	7.3 (2.3)	6.6 (3.2)	0.75
FEV1/FVC (%)	82.2 (9.3)	74.5 (17.9)	0.69
CRQ	87.8 (26.3)	76.0 (34.4)	0.34

VO_2_peak, peak oxygen uptake; Wpeak, peak workload; 6MWD, 6 min walking distance; MIP, maximum inspiratory pressure; TLC-total lung capacity; FVC, forced vital capacity; PEF, peak expiratory flow volume; FEV1, forced vital capacity; CRQ, Chronic respiratory questionnaire. ^*^p-value of Mann–Whitney-U-Test.

The Spearman correlation test revealed a significant correlation between CRQ and MIP in the NCS-negative group (*r*_*s*_ = 0.59, *p* = 0.02) but not in the NCS-positive group (*r*_*s*_ = 0.71, *p* = 0.14). 6MWD and MIP were significant correlated in the NCS-positive group (*r*_*s*_ = 0.88, *p* = 0.03) but not in the NCS-negative group (*r*_*s*_ = 0.062, *p* = 0.82). CRQ and 6MWD were not significantly correlated in both the NCS-negative and NCS-positive groups (*r*_*s*_ = 0.43, *p* = 0.09 and *r*_*s*_ = 0.6, *p* = 0.24, respectively).

### Discussion

In our explorative study of post COVID patients with persisting exercise intolerance and exertional dyspnea we identified in 27.1 % of the patients an abnormal phrenic nerve CMAP while the nerve conduction was normal. A reduced CMAP can reflect pathological changes in the muscle itself or can reflect axonal loss. In our findings axonal loss is not likely as phrenic nerve latency is normal. In the presence of axonal loss, some degree of slowing of the NCV can be expected, because axonal loss typically affects primarily the fast-conducting fibers and therefore NCV should be at least mildly decreased. Therefore, we suggest that the reduced CMAP in our patients is due pathological changes in the muscle itself.

The course of the acute disease was mild in our six patients, therefore critical ill myopathy can be excluded whereas systemic inflammation and immune cell infiltration to the muscle cells may be responsible for muscle fiber pathology leading to reduced muscle fiber contraction capacity. So, this may be seen as a mild type of myopathy and could be one factor in the pathogenesis of the persistent respiratory symptoms after COVID-19 Infection, which cannot be solely explained by decreased lung function parameters.

Recently abnormal inspiratory muscle weakness with upregulated neuro-ventilatory activity was assessed up to several months after COVID-19 infections, even in patients with mild diseases. Prolonged dyspnea in these patients were associated with exercise intolerance and decreased inspiratory muscle force (6, 24).

Interestingly, while lung function and exercise performance parameters do not differ between patients with normal and abnormal phrenic nerve amplitude. MIP, CRQ and 6MWD are closely interrelated within the NCS positive but not within the NCS negative group. Thus, the interrelation between these variables might be a consequence of the reduced CMAP of the phrenic nerve, indicating reduced inspiratory muscle force, walking performance and quality of life.

Patients after COVID-19 infection are suffering of prolonged dyspnea not explained by long-term pulmonary abnormalities and often only slightly decreased or even normal exercise capacity. In 100 patients 3–6 months after COVID-19 infection a higher ventilatory demand during exercise could be found expressed by high ventilatory equivalents especially for oxygen at submaximal exercise intensity (6). Moreover, a decreased diffusions capacity could be found in 37 % of these patients without changes in the CT scan of the lung (6). Respiratory muscle weakness could be one of the reasons for these functional adaptations to exercise. The pathogenesis of these persistent symptoms is largely unknown. Various hypothesis has been proposed such as diaphragm fatigue, acute inflammatory sensory and motor polyneuropathy (12, 13). Neuromuscular problems are common complications after prolonged intensive care hospitalization, affecting among 40% of all patients with prolonged duration of the muscle action potential and slowing of motor conduction velocities causing muscle weakness (16, 17). In patients with COVID-19 infections the polyneuropathy is assumed to be one of the possible mechanisms for respiratory muscle weakness (15).

To our knowledge, we presented one of the first explorative study demonstrating decreased phrenic nerve CMAP in patients recovering from COVID 19 with persisting symptoms without signs of neuropathy, due to normal nerve conduction.

As the pathomechanisms for ongoing dyspnea in post-COVID patients are still not fully understood, diaphragm impairment has been assessed in prior studies (26). Previous case reports were able to prove phrenic nerve palsy in patients with unilateral or bilateral diaphragm weakness (27). In our patient collective phrenic nerve neuropathy was not present indicating that neuropathy of the phrenic nerve might not be a common finding in patients with ongoing dyspnea after mild COVID symptoms.

Several studies are addressing patients after severe COVID-19 disease or patients hospitalized during COVID-19 infection (28, 29). Direct viral infiltration has been proven in histologic specimen also showing degeneration of myofibres and muscular dystrophy. Further Shi et al. was able to demonstrate myopathy and increased expression of fibroblast growth factor as well as accumulating fibrosis (30). Soares et al. (16) is supporting those findings as they were able to prove degenerating myofibres and 2-folds higher fibrosis rate in COVID-ICU patients compared to ICU patients. Myopathic changes in critically ill patients are a finding not necessarily associated with SARS-CoV-2 itself. Knowledge is still lacking in patients reporting ongoing exertional dyspnea after mild COVID 19 without hospitalization.

A reduced CMAP can reflect pathological changes in the muscle itself or can reflect axonal loss. In our findings axonal loss is not likely as phrenic nerve latency is normal. In the presence of axonal loss, some degree of slowing of the NCV can be expected. Axonal loss affects primarily the fast-conducting fibers and therefore NCV should be at least mildly decreased. Moreover, the configuration of the CMAP was normal which is seen in myopathy, but not in neuropathy. Therefore, we suggest that the reduced CMAP in our patients is due pathological changes in the muscle itself. In addition, we showed an association between the decreased CMAP of the phrenic nerve to a lower inspiratory muscle force and reduced exercise capacity. This reduced muscle fiber contraction capacity due to muscle fiber pathology could be one factor in the pathogenesis of the persistent respiratory symptoms after COVID-19 Infection, which cannot be solely explained by decreased lung function parameters.

Limitations of our study is the small sample size due to the explorative setting with a high standard variation in the small group of decreased phrenic nerve amplitude. Therefore, it could only be shown a tendency of differences between both groups without reaching significance. Moreover, we did not perform surface EMG of needle EMG, which would give more information about the pathophysiology of the diaphragm. Another limitation is that the lack of significant associations between MIP, CRQ and 6MWD in those with unaffected CMAP cannot be explained in this study but only hypothesize by the fact that exercise limitation is due to many different causes such as deconditioning, co-morbidities, and medication. Due to the explorative character of the study and the small sample size, no correction was done for possible cofounders.

## Conclusions

The present findings of this explorative study suggest that respiratory muscle weakness and exercise capacity is associated with reduced phrenic nerve CMAP without signs of neuropathy. This indicates that muscle fiber pathology of the diaphragm may be one pathophysiological factor for the prolonged respiratory symptoms after COVID-19 infections. Larger observational and longitudinal studies are necessary for better understanding of the underlying mechanisms.

## Data availability statement

The original contributions presented in the study are included in the article/supplementary material, further inquiries can be directed to the corresponding author.

## Ethics statement

The studies involving humans were approved by the Ethic Committee of the Medical University of Vienna. The studies were conducted in accordance with the local legislation and institutional requirements. The participants provided their written informed consent to participate in this study.

## Author contributions

KV: Writing – original draft, Writing – review & editing. HN: Writing – review & editing. RZ: Writing – review & editing. JM: Writing – review & editing. BS: Writing – review & editing. GB: Writing – review & editing. JF: Writing – review & editing. LY: Writing – original draft, Writing – review & editing. MB: Writing – original draft, Writing – review & editing. TP-S: Writing – original draft, Writing – review & editing. MP: Writing – review & editing.
